# Lower lip squamous cell carcinoma in patients with photosensitive disorders: Analysis of cases treated at the Brazilian National Cancer Institute (INCA) from 1999 to 2012

**DOI:** 10.4317/medoral.21960

**Published:** 2017-12-24

**Authors:** Jorena-Fraga-Pimenta Borges, Nattália-Di Lanaro, Vagner-Gonçalves Bernardo, Rodolpho-Mattos Albano, Fernando Dias, Paulo-Antônio-Silvestre de Faria, Luis-Felipe-Ribeiro Pinto, Simone-de Queiroz-Chaves Lourenço

**Affiliations:** 1 DDS, Programa de Residência Multiprofissional em Odontologia Oncológica. Instituto Nacional de Câncer (INCA); 2 DDS, Programa de Carcinogênese Molecular - CPQ, Instituto Nacional de Câncer (INCA); 3 PhD, Departamento de Bioquímica, Universidade do Estado do Rio de Janeiro (UERJ); 4 PhD, Departamento de Cirurgia de Cabeça e Pescoço, Instituto Nacional de Câncer (INCA); 5 MD, Divisão de Patologia, Instituto Nacional de Câncer (INCA); 6 PhD, Programa de Carcinogênese Molecular - CPQ, Instituto Nacional de Câncer (INCA); 7 PhD, Programa de Pós-Graduação em Odontologia, Universidade Federal Fluminense (UFF)

## Abstract

**Background:**

Lower lip squamous cell carcinoma (LLSCC) is a common malignancy of the head and neck, being mainly a consequence of a chronic exposure to ultraviolet (UV) light solar radiation. Here, we evaluated the clinicopathological profile of patients with photosensitive disorders (xeroderma pigmentosum, lupus erythematosus and albinism) that developed LLSCC.

**Material and Methods:**

Data from patients who had a diagnosed LLSCC with a prior xeroderma pigmentosum, lupus erythematosus or albinism diagnosis that were treated at INCA from 1999 to 2012 were collected from patients’ medical records (n=16). The control group was composed of 68 patients with LLSCC without a medical history of photosensitivity. The clinicopathological data of this study population were collected and the association between these variables was analyzed by Fisher’s exact test. Survival curves were constructed using the Kaplan–Meier method and compared by log-rank test. All statistical analyses were performed using SPSS statistics package.

**Results:**

The mean age of patients in the photosensitive and non-photosensitive groups was 42 years and 67 years, respectively (*p*<0.0001). A previous history of malignant diseases was more common in the photosensitive group (*p*=0.001). In both groups, most tumors showed a pathological stage I/II disease. Overall and cancer-specific survival were not statistically different. However, disease-free interval showed a significant difference (*p*=0.01) between the photosensitive and non-photosensitive patients.

**Conclusions:**

Photosensitive patients presented LLSCC at earlier age but it usually was not the primary tumor in these patients. Furthermore, a more aggressive pathological behavior was not seen when compared with tumors from non-photosensitive patients. The disease-free interval was lower in photosensitive patients, as expected.

** Key words:**Lip cancer, Xeroderma Pigmentosum, Albinism, Lupus erythematosus.

## Introduction

Cancer of the lip and oral cavity accounted for 300,000 cases in 2012 (2.1% of the world’s total) (1). In Brazil, data from INCA (Brazilian National Cancer Institute) indicate that oral cancer is the fifth most common cancer among males and the twelfth among females, with 15,490 new cases expected in 2016 (2).

Lip cancer arises at the junction between the oral cavity and the skin, appearing predominantly in white men. The incidence peak for lip cancer is in the seventh decade of life. More than 90% of malignant tumors of the lip are squamous cell carcinomas (SCC) (3).

Lip squamous cell carcinoma is one of the most common malignancies of the head and neck, comprising about 12% of all cancers in this region and 25% of oral cavity cancers. The disease is usually diagnosed at an early stage, which contributes to a better prognosis (4-6).

Lip cancer is mainly a consequence of a chronic exposure to ultraviolet (UV) light solar radiation, especially UVB, with the lower lip being the affected site in 80% of the cases (7). In Brazil, lip cancer is a significant health problem because this tropical country has a high incidence of UV radiation (8).

The main risk factors associated with the development of lower lip SCC (LLSCC) are outdoor activities, however, sociodemographic factors and lifestyle, immunosuppression, tobacco and alcohol consumption, and even genetic susceptibility may produce a synergistic effect (7). In addition, an oral potentially malignant disorder (actinic keratosis) increase lip cancer risk (9).

In 2017, the WHO classification of Head and Neck Tumors described the oral potentially malignant disorders as generalized clinical entities associated with a significantly increased risk for SCC. These include erytroplakia, erytroleukoplakia, leukoplakia, oral submucous fibrosis, dyskeratosis congenita, smokeless tobacco keratosis, chronic candidiasis, lichen planus, discoid lupus erythematosus, syphilic glossitis and actinic keratosis (lip only) (9).

Here, we evaluated the clinicopathological profile of patients with xeroderma pigmentosum, lupus erythematosus or albinism that developed lip SCC and were treated at INCA between 1999 and 2012.

## Material and Methods

-Study Design

This study was a retrospective and descriptive analysis of LLSCC treated at the Head and Neck Surgery Department from 1999 to 2012. We identified five patients with LLSCC and albinism, four patients with LLSCC and lupus erythematosus and seven patients with LLSCC and xeroderma pigmentosum (16 patients with a prior photosensitivity disorder). Another 68 patients with LLSCC and without photosensitivity disorders were selected for comparison. Patients were paired by gender. Clinicopathologic information on each case, including age, alcohol and tobacco use, pathologic stage (pTNM), grade of tumor differentiation (9), previous history of cancer before lower lip cancer diagnosis, presence or absence of tumor recurrence, and patient survival were obtained from medical records and tumor registries. The inclusion criterion was the availability of medical records. This study was reviewed and approved by the institutional ethics committee (CEP125/10).

-Statistical Analysis

Statistical analyses were performed using SPSS statistics package for Windows (version 20.0, IBM Corp., Armonk, NY, USA). The association between clinicopathological variables was analyzed by Fisher’s exact test or Chi-square test. Survival analysis was done using the Kaplan–Meier method (the log-rank test was used to compare survival curves). Tests were considered statistically significant when the *p*-value was <0.05.

## Results

Eighty-four patients were included in this study and their clinicopathological characteristics are shown in  Table 1.

**Table 1:**
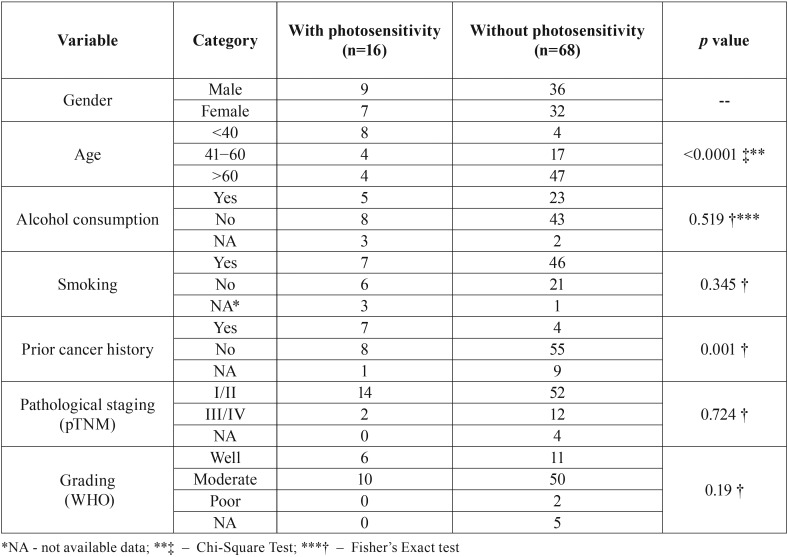
Clinicopathological features of patients with LLSCC, with and without photosensitivity, treated at INCA from 1999 to 2012.

There was a significant difference between the mean age at lower lip cancer diagnosis in the photosensitive group (42 ± 22.8 years, range: 11 – 77 years) and non-photosensitive group (66.7± 14.4 years, range: 32 – 99 years, *p* = 0.001). ln the photosensitive group, a positive history of previous malignancy was more common when compared with the non-photosensitive group (*p*= 0.001). No significant difference was observed regarding both alcohol consumption (*p*= 0.519) and tobacco smoking (*p*= 0.345).

The pTNM showed that most cases were either stage I or II, comprising 14 cases (87.5%) in the photosensitive group and 52 cases (81.2%) in the non-photosensitive group. There was no difference in the pathological staging between the two groups (*p* = 0.742). Most cases were moderately differentiated tumors and there was no significant difference between the groups (*p*=0.19).

The 5-year OS, CSS, and DFI rates were 57.4%, 57.4% and 37.4%, respectively, in the photosensitive group. The rates for the non-photosensitive group were 49.1% (OS), 52.6% (CSS) and 52.4% (DFI) ( Table 2).

**Table 2:**
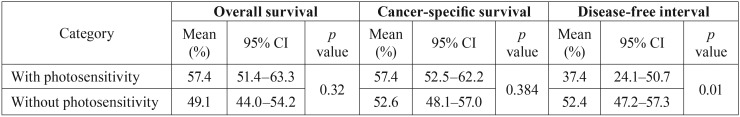
Survival in patients with and without photosensitivity.

**Figure 1:**
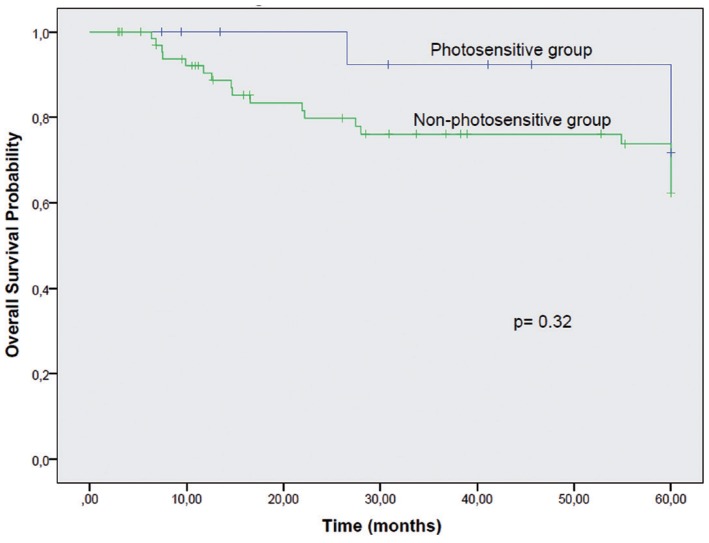
Overall Survival. Kaplan-Meier curves for Photosensitive and Non-photosensitive Groups according to overall survival, with no significant difference between the curves (*p*=0.32).

**Figure 2:**
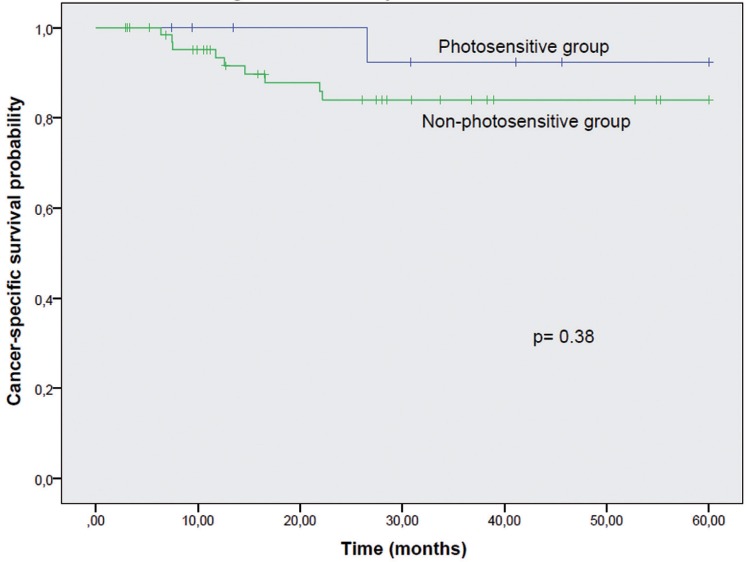
Cancer-specific Survival. Kaplan-Meier curves for Photosensitive and Non-photosensitive Groups according to cancer-specific survival, with no significant difference between the curves (*p*=0.384).

**Figure 3:**
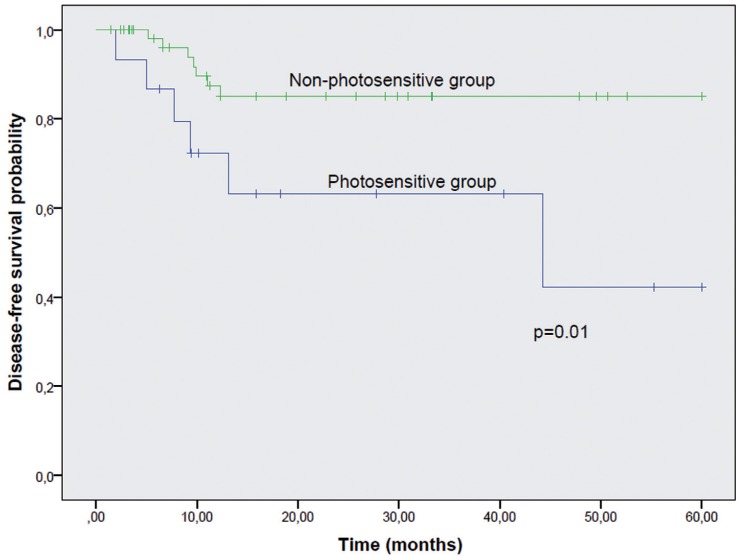
Disease-free interval. Kaplan-Meier curves for Photosensitive and Non-photosensitive Groups according to disease free interval. The presence of a photosensitive disorder indicated a lower probability of disease free survival (*p*=0.01).

Overall survival (*p*=0.32) and cancer-specific survival (*p*=0.384) were not different between the two groups (Figs. 1,2). However, a significant lower disease-free interval was seen between photosensitive and non-photosensitive patients (*p*=0.01) (Fig. 3).

## Discussion

This paper describes a case series of 16 patients with photosensitive disorders that developed LLSCC in a tertiary cancer reference center. They were paired by gender because there is a clear female predominance for lupus erythematosus (9-11).

The comparison between lower lip tumors from patients with and without photosensitivity showed a difference regarding the age at lip cancer diagnosis, with patients with photosensitivity developing lip cancer at a much earlier age (*p*<0.0001) but, generally, this cancer was not the primary tumor developed by these patients.

Both groups had similar frequencies of tobacco and alcohol consumption, but medical records did not allow an accurate assessment of occupational UV-light exposure.

A previous cancer history was also significantly associated with the photosensitive patients (*p*=0.001). Other groups had already shown the development of LLSCCs in albino patients previously affected by skin cancer (12,13), and Nico and coworkers stated that patients with LE may present previous tumors in malar, ear and scalp regions (14).

Most patients had an initial pathological stage I/II disease, irrespective of their photosensitive condition. The same is observed in published series of patients without photosensitivity. Kornevs *et al.* (15) and Souza *et al.* (8) showed that most patients without photosensitivity also had a pathological stage I/II disease (84.4% and 76.7%, respectively). This ‘early stage characteristic’ shows that LLSCC are less aggressive malignancies when compared with intra-oral lesions 16, even in patients with a prior history of diseases related to photosensitivity.

Tumors in our study were mostly classified histopathologically as moderately differentiated (86.9%). Géraud *et al.*, assessing patients without a previous history of photosensitive disorders found that 56% of LLSCC were moderately differentiated (17). Other studies such as Souza *et al.* and Biasoli *et al.* observed a predominance of well differentiated tumors, assessing patients without photosensitivity (8,18). In a study performed in Zimbabwe (19), well and moderately differentiated LLSCC were equally diagnosed in albinos, and patients with xeroderma pigmentosum developed mostly well-differentiated LLSCCs. Another study evaluated the malignant potential of chronic discoid lupus erythematosus, and patients that developed LLSCC had mostly well differentiated tumors (10).

The disease-free interval was statistically lower in photosensitive patients (*p*=0.01) but cancer-specific survival showed that LLSCC did not influence survival (*p*=0.384). In our study, most photosensitive patients were alive after the five-year follow-up period.

The disease-free interval was lower in photosensitive patients, although the overall survival, cancer-specific survival and pathological stage are not different between both groups. These tumors do not show a more aggressive behavior when compared with tumors from non-photosensitive patients.

This paper reinforces the association between photosensitive disorders and LLSCC development, although the albinism and xeroderma pigmentosum were not cited as an oral potentially malignant disorder by the WHO (2017), its importance must be considered.
